# Evolutionary game theory for physical and biological scientists. II. Population dynamics equations can be associated with interpretations

**DOI:** 10.1098/rsfs.2014.0038

**Published:** 2014-08-06

**Authors:** David Liao, Thea D. Tlsty

**Affiliations:** Department of Pathology, University of California San Francisco, San Francisco, CA 94143, USA

**Keywords:** game theory, modelling, stromal–epithelial interactions, cell–cell interactions, tissue microenvironment, biomedical engineering

## Abstract

The use of mathematical equations to analyse population dynamics measurements is being increasingly applied to elucidate complex dynamic processes in biological systems, including cancer. Purely ‘empirical’ equations may provide sufficient accuracy to support predictions and therapy design. Nevertheless, interpretation of fitting equations in terms of physical and biological propositions can provide additional insights that can be used both to refine models that prove inconsistent with data and to understand the scope of applicability of models that validate. The purpose of this tutorial is to assist readers in mathematically associating interpretations with equations and to provide guidance in choosing interpretations and experimental systems to investigate based on currently available biological knowledge, techniques in mathematical and computational analysis and methods for *in vitro* and *in vivo* experiments.

## 1. Introduction

**Figure 1.**
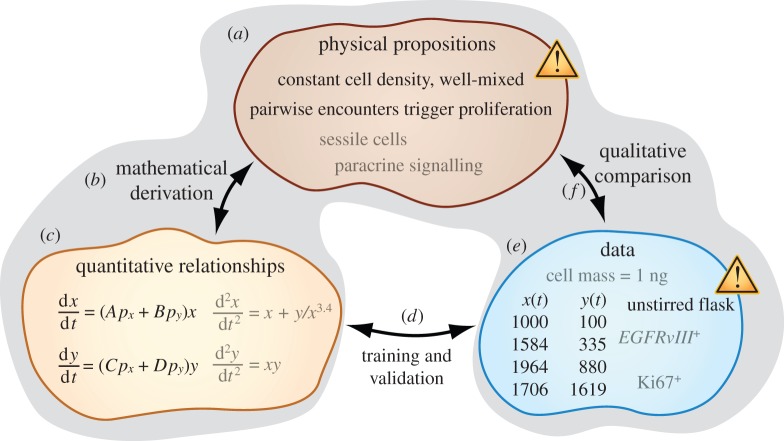
Overview of physical sciences modelling as also illustrated in [1]. In the preceding manuscript, we illustrated how fitting functions can be (*d*) compared with (*e*) experimental time-course population measurements. The focus of this manuscript is highlighted in grey. In this manuscript, we give examples of how (*a*) physical models are used (*b*) to mathematically derive (*c*) fitting equations. We also discuss biological knowledge of experimental systems that can help (*f*) to identify choices of candidate propositions. For example, if a provisional model relies on an assumption of thorough mixture and cell–cell contact, but the cellular population under study is not motile and is cultured in an unstirred flask, direct observation at the microscopic scale and physical propositions disagree (alert pictograms), initiating a search for alternative propositions (and, potentially, fitting equations). (Online version in colour.)

In an accompanying manuscript [1], we present a method for using time-course measurements to train and to validate a set of differential equations1.1
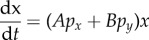
1.2
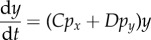
describing the dynamics of interacting populations. This procedure provides an ability to reject equations that are inconsistent with data. Performing this skill increases our confidence in predictions obtained from those equations that do validate. Nevertheless, even when equations agree with data, model assumptions may yet prove false. In other words, models and equations are distinct. A statistician might use the term ‘model’ to refer to a parametrized equation, and, in this parlance, a set of validated differential equations might be considered a ‘model’. However, *taken in isolation, such training and validation do not constitute* physical *modelling***.** A physicist uses the term ‘model’ to refer to a set of physical propositions (‘assumptions’). For a physicist, modelling is more than writing down an equation that imitates a plot of data. As illustrated in figure 1, which also organized the previous manuscript, physical modelling is the development of a consistent set of physical propositions, mathematical equations and data. The previous manuscript only addressed the process of training and validating equations using data, as highlighted in figure 1*d*. In this second tutorial, we provide examples of how differential equations can be associated with models using mathematical derivations, as highlighted in figure 1*f*. In other words, the focus of this manuscript is to demonstrate how equations can be associated with interpretations.

### 1.1. Manuscript organization

We first describe in §2 a common way that evolutionary game theory (EGT) models are misinterpreted. This confusion arises from the fact that the phrase ‘game theory’ is used both in economics and in biology. In §3, we show how EGT models can be properly associated with microscopic hypotheses using mathematical derivations. The value of performing a derivation is not only to rationalize the use of a set of equations to describe an experiment. Another benefit is to demonstrate that a set of equations can be consistent with multiple sets of propositions. This means that no particular set of propositions should be accepted as ‘true’ merely because it is consistent with a set of equations that is, itself, consistent with data. With so many models possibly associated with a particular set of equations, we seek strategies for narrowing down the list of candidate interpretations on which to focus in experimental studies. In §4, we outline how choices of models, equations (or simulations) and experiments can be refined by considering biological knowledge of cell–cell interaction processes, analysis and computer simulation techniques from the physical sciences, and availability of cell culture, manipulation, and imaging techniques from biology, physics and engineering. In the Discussion, we describe opportunities for advancing the application of game theory in biology, not only through experimental technology development, but also by expanding our concept of biological systems beyond the concept of ‘systems of cells’. We conclude with the possibility that our understanding of the essential features of biological populations might one day be informed by a deeper understanding of dissipative structures.

## 2. Evolutionary game theory does not require that cells display sophisticated intelligence

Shared use of game theoretic terminology in economic and biological applications leads to misinterpretation of the application of EGT to cellular population dynamics. In this section, we clarify this confusion. In the previous manuscript, we used the phrase ‘EGT’ to refer to models of cell interactions in which net expansion rates depended on the proportions at which different cell types are found in an overall population. This definition might be surprising for readers previously introduced to applications of game theory in economics. It is important to be aware that economists, biologists, computer scientists and physical scientists use game theoretic terminology in a variety of ways. For the purposes of this tutorial, game theory broadly encompasses the reasoning and conclusions that follow (i) from investigating how the *outcome* (e.g. ability to reproduce, paycheck, jail time, abstract utility, etc.) of an individual agent (e.g. cell, businessperson, criminal, snowplough driver, etc.) depends on the status of other agents with which the individual interacts. Game theory also includes the reasoning and conclusions that follow (ii) from investigating how an agent's *actions* can be influenced by social context.

As illustrated using the two examples in table 1, these concepts can be applied to systems composed of rational decision makers and also to populations of robotically replicating cells. In the first example, a businessperson can draw a table (pay-off matrix) listing the profit anticipated in various scenarios that correspond to different combinations of her possible business strategies and the possible strategies of her competitors. By analysing her pay-off matrix, the businessperson then chooses a strategy expected, on average, to maximize her return. If the pay-off matrix changes, the businessperson can study the new matrix to determine whether a different strategy would now optimize her expected return. This is an example of game theory applied to ‘comparative statics’: optimal strategies are identified for different pay-off matrices, but dynamic processes by which the pay-off matrices or business strategies are changing over time are not explicitly described. In a more biologically oriented example, individual cells in an ecosystem might display net replication rates that vary with the relative proportions of different cell types in the environment. While the individual cells might lack the intelligence needed to write down and scrutinize a pay-off matrix, the proportions of cells expressing different phenotypes in the population *as a whole* can nevertheless be altered owing to natural selection operating over generations of reproductive competition. The study of such evolutionary dynamics is referred to as ‘EGT’. Typically, the individual cells are called replicators, and the parameters in ‘replicator dynamics’ equations are often formatted in tables also called pay-off matrices.

**Table 1.** Game theoretic concepts in the analysis of comparative statics and evolutionary dynamics.

	outcome for an agent depends on its own strategy and strategies of other agents	distribution of strategies that agents display is influenced by strategies of agents with which they interact	solution condition
comparative statics	a pay-off matrix is a table of pay-offs (utilities) an agent is anticipated to receive in different scenarios	an agent can adopt a strategy that maximizes pay-off by comparing numerical entries in a pay-off matrix. Changing the problem by changing the pay-off matrix can change the ‘best’ strategies agents can adopt	interacting agents adopt a set of strategies so that no agent can increase her pay-off by unilaterally changing her strategy
evolutionary dynamics	net cell population expansion rate is a function of the demographic composition of the cells in the environment	in many models, *individual agents* lack the intelligence needed to scrutinize entries in a pay-off matrix. Less fit cells do not ‘know’ to quit. Nevertheless, a *population* can become relatively pruned of less fit cell types over generations of reproductive competition	relative proportions of different cell types in a population achieve a stable (homeostatic, tends to self-restore) steady state

Owing to the similar language associated with the economically oriented and biologically oriented examples we have just described, it is common for the application of EGT to be misunderstood as relying on an unrealistic and restrictive assumption that cells rationally choose their behaviours [2,3]. Such misunderstanding can be exacerbated by the fact that rational agents described by comparative statics and cellular populations described by EGT are sometimes said to share the same solutions. The way in which these populations achieve ‘the same’ solutions is described in the electronic supplementary material. The connection has the flavour of an abstract mathematical relationship, rather than of a profound philosophical conclusion that implies sophisticated intelligence in cells.

## 3. Mathematical derivation of replicator dynamics equations

In §2, we demonstrated that relying primarily on word models and labels alone can lead to misinterpretation of biological applications of EGT. Even though the term ‘EGT’ includes the word ‘game’, using EGT does not suppose the presence of ‘game players’ possessing humanly sophisticated intelligence and will. Given that it is difficult to associate interpretations with equations from EGT using word models alone, it is helpful to understand how mathematical derivations can be applied to accomplish this goal. In this section, we provide five examples of how EGT population dynamics equations can be obtained by quantitatively expressing assumptions about microscopic cell–cell interaction processes. The first three examples show how equations (1.1) and (1.2) can be obtained by proposing that true-breeding is triggered by cell–cell collisions, by proposing that cell-type conversion is triggered by cell–cell collisions or by proposing that cells modulate their proliferation rates by taking a census of their environment. The final two examples show how a more general version of equations (1.1) and (1.2) can be obtained by assuming that more than two cells can collide or, for example, by assuming that cell surface receptor interactions display cooperativity. Together, these examples illustrate that a variety of models can be associated with a set of equations, so that empirical validity of a particular set of equations cannot be regarded as proof of truth of any particular model.

### 3.1. Cell replication triggered by pairwise cell–cell collisions

**Figure 2.**
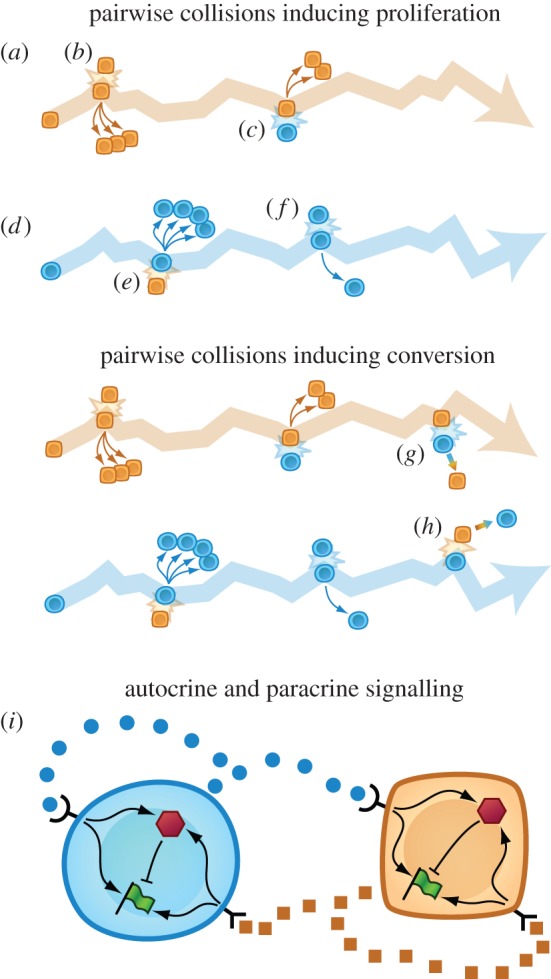
Different physical propositions can lead to the same fitting equations. (*a*) Trajectory of a focal individual of type *x* (square, yellow cell), showing a collision (*b*) with another cell of type *x* and a collision (*c*) with another cell of type *y* (round, blue cell). (*d*) Trajectory of a focal individual of type *y* showing a collision (*e*) with another cell of type *x* and a collision (*f*) with another cell of type *y*. Each of these collisions triggers the focal individual to produce progeny. Two additional possibilities are (*g*) that the square, yellow focal individual can collide with a round, blue cell, causing that round, blue cell to convert to the square, yellow state, and (*h*) that the round, blue focal individual can collide with a square, yellow cell, causing that square, yellow cell to convert to the round, blue state. (*i*) Alternative model consistent with the same dynamics equations obtained from (*a*–*h*). Cells communicate through signalling factors that activate the synthesis of both intracellular proliferation and anti-proliferation signalling molecules. The molecular circuit topology in each cell is a pair of IFFLs that share an inhibitory edge. (Online version in colour.)

While the concepts illustrated in this and the following examples are applicable to systems with arbitrary numbers of populations, we will continue to use two-population systems, as was the focus in the previous manuscript, to simplify illustrations and equations. Our first example shows how equations (1.1) and (1.2) can be obtained by proposing that cells trigger each other to proliferate through pairwise collisions. In figure 2, a (square, yellow) cell of type *x* follows the trajectory in (*a*), while a (round, blue) cell of type *y* follows the trajectory in (*d*). The cell whose trajectory is tracked in each diagram is called the ‘focal individual’. We will calculate the number of progeny produced by each focal individual using the following propositions.

We propose that the co-culture is thoroughly mixed to ensure that each cell encounters a fixed number of other cells per unit time. We label this rate *r*. In other words, over a time interval Δ*t*, each cell collides with *r*Δ*t* other cells. In a fraction of these collisions, the focal individual encounters a cell of type *x*. In the remaining fraction of collisions, the focal individual encounters a cell of type *y*. We propose that the cell culture is mixed with such vigour that these fractions equal the population fractions *p_x_* and *p_y_*, respectively. Finally, we propose that each collision triggers the focal individual to replicate and, thus, to contribute progeny to the overall population. To be specific, a collision between the square, yellow focal individual and a square, yellow cell at (*b*) triggers the focal individual to produce *A*/*r* additional square, yellow cells. Here, *A*/*r* = 3 cells. The collision at (*c*) is different because the focal individual collides with a round, blue cell, rather than with a square, yellow cell. This collision triggers the focal individual to contribute *B*/*r* square, yellow cells to the population. Here, *B*/*r* = 2 cells. The propositions we have listed describe (i) the number of cells with which a focal individual collides during an interval of time, (ii) the fraction of these collisions that involve a cell of type *x* (as well as the fraction that involve a cell of type *y*), and (iii) the number of progeny that the focal individual produces as a result of each type of collision. Multiplying these factors for each type of collision and then adding the resulting products gives the total number of progeny contributed by the focal individual3.1
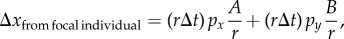
during the illustrated time interval. Note that factors of *r* cancel. Because the focal individual in (*a*) is representative of each cell of type *x* in the co-culture, we multiply the quantity in equation (3.1) by the number of cells of type *x* to obtain the total number of progeny of type *x* added to the population during time interval Δ*t*. Dividing out the time interval Δ*t*3.2
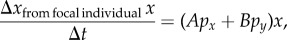
we obtain a rate of change consistent with equation (1.1). Equation (1.2) can be derived by analysing figure 2*d*–*f* in the same way as we have analysed panels (*a*–*c*). We have derived equations (1.1) and (1.2) by proposing that cells breed true and replicate upon collision. It is important to recognize that other physical propositions can also lead to the same equations. In §3.2, we show that equations (1.1) and (1.2) do not require cells to breed true, and in §3.3, we show that equations (1.1) and (1.2) do not even require cell–cell collisions.

### 3.2. Horizontal gene transfer triggered by cell–cell collisions

Here, we show that equations (1.1) and (1.2) are consistent with the dynamics of cells that do not breed true. In other words, the progeny produced by a cell of type *x* could be of type *y* and *vice versa*. We begin by re-expressing equations (1.1) and (1.2) using slightly different parameter names3.3
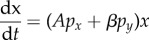
3.4
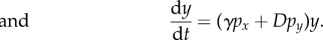


Without loss of generality, we introduce a constant *φ*, we let 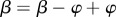 and 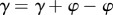, and we then define 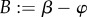 and 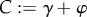 to obtain3.5
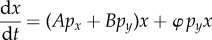
3.6
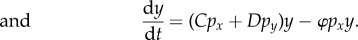


Letting 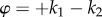 and observing that *p_x_ y* = *p_y_ x*, we finally obtain3.7
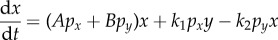
3.8
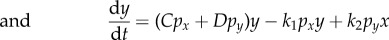
differential equations in a format consistent with socially induced cell-type conversion. The term *k*_1_*p_x_y* can be interpreted as a rate at which cells of type *y* become cells of type *x*. This rate increases in proportion to the number of cells of type *y* available to become cells of type *x*. The proportionality coefficient includes a constant factor *k*_1_, as well as a copy of *p_x_*, indicating that the frequency of this process increases with increasing proportion of cells of type *x* in the environment. This would be consistent with cell-type conversion triggered by cell–cell contact. For example, if the distinction between square, yellow and round, blue cells were genetic, such an event might occur as illustrated in (*g*). Here, the square, yellow focal individual collides with a round, blue cell, momentarily triggering the square, yellow cell to release an exosome containing genetic material that then integrates into the genome of the round, blue cell. The term *k*_2_ *p_y_x* could be interpreted in terms of the analogous conversion in the opposite direction, as illustrated in (*h*). Here, the round, blue focal individual collides with a square, yellow cell, causing the square, yellow cell to convert to the round, blue state.

The mechanism we have described in this subsection is just one example of a way that cell-type conversion could occur as a result of cell–cell contact. In the illustrations in (*g*) and (*h*), two cells having different cell types approach each other, and one cell type is abandoned. Another possibility is that not just one but both of the cell types initially present become lost. This can happen when two cells fuse to become a third cell whose genetic make-up is distinct from the genetic state of both colliding cells, as observed in metastatic mammary cells [4].

### 3.3. Autocrine and paracrine signalling

A feature common to the models in §§3.1 and 3.2 is the proposition that cell–cell collisions can trigger changes in the numbers of cells of type *x* and *y*. However, interactions that affect population dynamics (e.g. affecting drug sensitivity) can also occur between physically separated cell populations [5]. Here, we describe a third model that also leads to equations (1.1) and (1.2), but without the assumption that cells display the mobility that would be needed to sustain ongoing cell–cell collisions.

In figure 2*i*, both square, yellow and round, blue cells emit signalling molecules. If we suppose that the molecules are well-mixed and short-lived, then the concentration of blue circular molecules is proportional to the immediate number of round, blue cells in the culture, and the concentration of yellow square molecules is proportional to the immediate number of square, yellow cells in the culture. The concentrations of signalling molecules correspond to the population sizes *right now* because the short lifespan of each signalling molecule ensures that molecules emitted by cells in the distant past have already degraded. We propose that each cell contains a molecular circuit consisting of a proliferation signal (green flag) that is degraded by a stop signal (hexagonal stop sign) in bimolecular collisions. As a gross simplification, we assume that a cell's propensity for proliferation is proportional to the number of copies of the proliferation signal it contains. We list additional assumptions as we develop an expression for the level of the proliferation signal in cells of type *x*. Suppose that, when cell surface receptors bind their cognate ligands, they can catalyse synthesis of the stop signal. If the stop signal is short-lived, its abundance in each cell of type *x*3.9
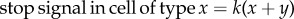
can be proportional to the current number of cells in the culture. Suppose that the bound state of the cell surface receptors also catalyses the synthesis of the proliferation signal so that the synthesis rate depends linearly on population *x* and linearly on population *y*, with proportionality coefficients *bkA* and *bkB*, respectively. Assuming that the abundance of the proliferation signal reaches steady state, each cell of type *x* should contain3.10
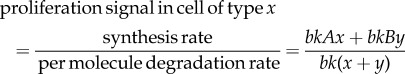
a level of proliferation signal in which *x* and *y* appear in both the numerator and the denominator [6]. Here, the rate coefficient for the bimolecular reaction that degrades the proliferation signal is *b*. This is equivalent to stating that the fitness of cells of type *x* is *f_x_* = *Ap_x_* + *Bp_y_*, which is, itself, equivalent to equation (1.1). Likewise, equation (1.2) can be obtained by applying to cells of type *y* the line of reasoning that produced equations (3.9) and (3.10).

Interestingly, the circuit architecture illustrated in (*i*) involves incoherent feed-forward loops (IFFLs). An IFFL consists of a node that activates two downstream nodes, one of which inhibits the other. For example, each cell surface receptor activates both the proliferation signal and the stop signal, and the stop signal inhibits the proliferation signal. Each cell in this cartoon contains a pair of IFFLs that share an edge. IFFL motifs are considered as possible tools for creating transient responses to step changes in external stimuli [7]. Based on the derivation in this subsection, it is possible that another biological function of IFFL motifs is to provide ongoing measurements of population fractions. To pursue this possibility, it could be profitable to investigate how often IFFLs are found to share common edges in molecular network diagrams.

**Figure 3.**
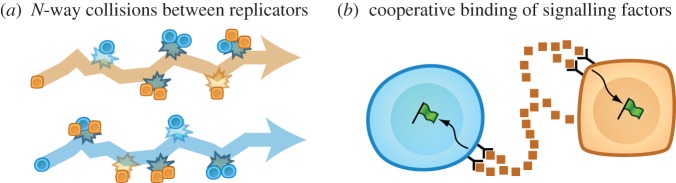
Additional examples of models that can be used to derive population dynamics equations. (*a*) Cell replication could be triggered by collisions involving more than two cells. (*b*) Cooperative binding of signal factors to cell surface receptors can lead to a sigmoidal relationship between cellular fitness (net replication rate) and the population fraction of cell type *x*. (Online version in colour.)

We have just provided three examples of physical models that lead to equations (1.1) and (1.2). These derivations would be relevant if equations (1.1) and (1.2) were validated for a particular biological system using the methods described in §3 of the previous manuscript [1]. However, if equations (1.1) and (1.2) failed to validate in a particular system, for example if the oscillatory trajectory originating from figure 3*h* of the previous manuscript [1] were observed, it would be necessary to develop another set of population dynamics equations. One of the reasons that equations (1.1) and (1.2) can fail is that they contain fitnesses, *f_x_* = *Ap_x_* + *Bp_y_* and *f_y_* = *Cp_x_* + *Dp_y_*, that are necessarily linear functions of population composition. In §§3.4 and 3.5, we describe two physical models that can be used to obtain population dynamics equations in which the fitnesses are, instead, more complicated *polynomial* functions of population composition. For brevity, we will present these two examples using only conceptual outlines, rather than detailed mathematical derivations.

### 3.4. *N*-way cell–cell collision dynamics

In figure 2*a–h*, we assumed that cells encountered each other only through pairwise collisions. In figure 3*a*, we relax this assumption by allowing for collisions between more than two cells at a time (examples involving 2, 3 and 4 cells illustrated). As a result, equation (1.1) is modified3.11
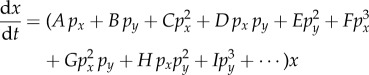
to include higher powers of the population fractions *p_x_* and *p_y_*. The fitness of cells of type *y* in equation (1.2) becomes a power series as well. It is beyond the scope of this tutorial to explain how such an equation can be trained and validated. However, we must emphasize to the novice in modelling that equation (3.11) and other equations that contain many fitting parameters are dangerous. They have a tendency to accommodate data even when the underlying physical models used for their derivation are inaccurate. As an example, consider the possibility that the dynamics of population *x* in a two-population system is well fitted by equation (3.11), specifically with the form3.12
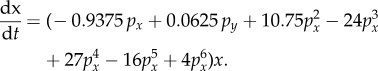
While this equation is consistent with an interpretation that proposes collisions involving pairs, triplets, quartets, quintets, sextets and septets of cells, it also has a suspicious property that the power series expression for the fitness can be approximated by a simple sigmoid3.13
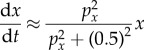
of Hill coefficient 2. Equation (3.13) can actually be derived using a simple cell-signalling circuit architecture described in §3.5, which does not assume any cellular collisions.

### 3.5. Cooperative binding of signal factors

In figure 3*b*, square, yellow cells (type *x*) and round, blue cells (type *y*) both present cell surface receptors that bind to a signalling factor released by cells of type *x*. Again, assuming short-lived signalling molecules, the concentration of square molecules is proportional to the current size of population *x*. Suppose that the total density of cells of type *x* and type *y* relative to the surrounding medium is constant (e.g. a biofilm in which the proteinaceous film and cellular mass expand in proportion). The concentration of square molecules is then also proportional to the population *fraction* of cell type *x*. Suppose that two cell surface receptors, at the same moment becoming bound, each to a copy of the cognate ligand, can dimerize and thereby trigger the synthesis of a proliferation signal. Finally, suppose that the dimer can break apart in a reaction that ejects the ligands from the binding pockets of both involved receptors. Taken together, this model is consistent with a fitness function that is a second-order Hill function, as in equation (3.13).

One danger in using equation (3.11) as a fitting function is that a ‘successful’, but difficult to interpret, fit, like equation (3.12), could result. The approximate equivalence of equation (3.12) to equation (3.13) might not be noted, leading to favouring a cell–cell collision interpretation when the possibility of receptor dimerization should also be considered. It should be noted that the mechanism of dimerization in this model was specific, and so not every receptor known to dimerize would satisfy the assumptions of this particular model. For example, while the TGF-β superfamily of ligands induce dimerization of type-II receptors (technically, tetramerization of two type-II receptors with two type-I receptors), the binding events might not proceed in the way described in this subsection. The final assembly consists of one TGF-β superfamily ligand, which suggests that the assembly of the dimer might not require simultaneous ligand activation of two individual type-II receptors [8]. As a second example, EGFR family receptors are also known to dimerize, and crystal structures of doubly ligated EGFR dimers have been obtained. Nevertheless, it has been reported that dimers can be activated with a single ligand [9].

The five examples in this section provide only a sample of ways to derive fitting functions to describe cellular interactions. For an additional example in yeast that takes into account molecular details of metabolic public goods production and consumption, we refer the reader to a phenomenological study by Wang & Goldenfeld [10]*.* Taken together, the examples from this section show that fitting equations can be derived from diverse physical propositions. This illustrates a trade-off between generalizability and specificity. On the one hand, a particular set of fitting equations might accurately describe population dynamics for a variety of biological systems with different molecular details, but, on the other hand, agreement between the fitting equations and any particular system does not uniquely implicate a particular physical model. Thus, consistency between data and equations and between equations and a physical model should only be regarded as confirming that a model has not yet been rejected, not as ‘confirming’ that a model is true.

## 4. Designing studies of population interactions

Now that we have demonstrated that a variety of interpretations can be associated with a particular set of equations (and thus, data), we seek strategies for narrowing down lists of candidate models to focus on in experimental studies. This is part of a broader question. In figure 1, we list candidate propositions, candidate equations and candidate experiments which can then be investigated to see which propositions, equations and experiments are consistent and informative. How can we obtain these candidate lists? In this section, we outline potentially helpful considerations. These include biological knowledge of processes that contribute to cell–cell interactions, physical sciences expertise in mathematical analysis and computer simulation, and technical limitations of cell-culture and imaging systems.

### 4.1. Biological knowledge

When choosing a shortlist of models, it can be helpful to include models based on existing biological knowledge. If a set of equations is validated by data, the biologically motivated propositions underlying models under consideration can point to additional experiments to probe the limits of the domain of validity of the equations. If the equations are rejected by data, the biological propositions can point to previous experiments that might need to be re-interpreted.

We describe a few examples of how biological knowledge can improve our ability to build on the cartoon models in §3. Cells communicate through a variety of cytokines [11]. Thus, even when population dynamics data are accommodated by assuming that a single molecule mediates an interaction, it might be important to target a variety of soluble factors or receptors in order to change the dynamics of a tissue system. This insight might not be evident from a mathematically correct, but biologically uninformed, fit of equations (1.1) and (1.2). To explore a second example, we note that the cells in the models in §3 interact *either* through cell–cell collisions *or* through the dispersion of soluble factors. However, the mechanisms most relevant for a particular biological tissue system could also involve a combination, or neither, of these processes. For example, stromal cells can increase drug resistance in cancer cells via both cell-adhesion-mediated and soluble-factor-mediated pathways [12]. Communication modalities other than cell–cell contact and soluble factors are also possible. Mammary carcinoma-associated fibroblasts (CAFs) deposit extracellular matrix (ECM) with altered orientational order, and exposure to this altered ECM promotes a mesenchymal phenotype in mammary epithelial cells even in the absence of CAFs [13]. In this example, the signalling factor is not soluble, but, instead, deposited in place. Direct contact is not required for the observed communication between CAFs and epithelial cells. Other chemical and physical properties of the ECM that can influence cell behaviour include stiffness and cross-linking [14].

**Figure 4.**
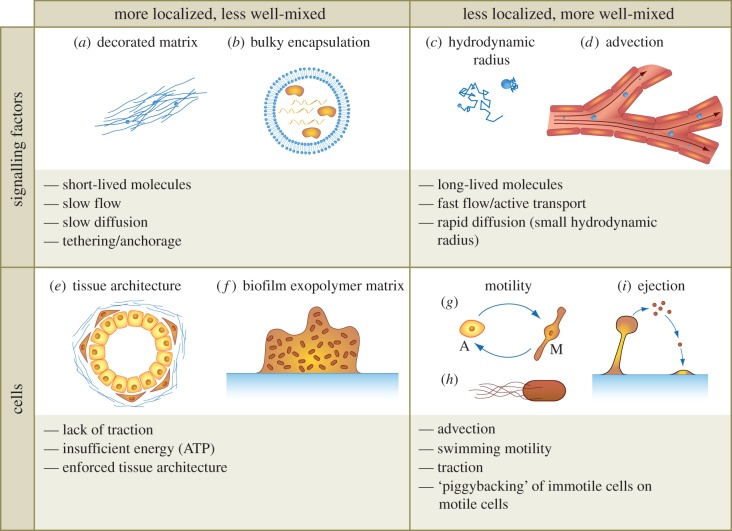
Factors that can contribute to localization versus homogeneous mixture in biological systems. Signalling factors might be relatively localized when (*a*) fixed on ECM or (*b*) encapsulated in bulky vesicles. (*c*) Smaller soluble factors or (*d*) factors carried by hydrodynamic flow could, in contrast, effect long-range cell–cell communication. Both (*e*) mammalian cell populations and (*f*) biofilm cellular communities display tissue architecture [17]. Transitions at the single-cell level (*g*) between amoeboid and mesenchymal phenotypes described in [18] might allow cells to move through spatially heterogeneous tissues. Socially coordinated phenotypic specialization can also contribute to spatial dispersal, as (*i*) when a cell population forms a stalk that supports a subpopulation of spore cells [19]. (Online version in colour.)

Detailed specification of the factors involved in cell–cell communication is not ‘just stamp collecting’ for the sake of adding *unnecessary* realism to models. In §4.2.1, we will discuss how spatially localized communication can influence the diversity that emerges in an ecology consisting of interacting subpopulations. Knowledge of the forms in which signals are delivered from cell to cell is helpful for understanding whether cell–cell interactions are localized or long-range. Exosomes provide an example. These microvesicles carry various cellular contents, including proteins, RNAs and miRNAs [15]. One difference between secreting proteins directly into the microenvironment and transporting proteins using exosomes is that exosomes have a hydrodynamic radius of approximately 40–100 nm, rather than a couple of nanometres [16]. The Stokes–Einstein relationship thus suggests that the mean-squared displacement through which a protein diffuses in a given time interval could be 20–50 times greater than the mean-squared displacement of the same protein encapsulated in an exosome. Figure 4 summarizes additional features of tissue architecture, cell motility and the physical properties of signalling molecules that can serve as clues to the spatial extent of cell–cell communication in a biological system. As an example of how these features can be combined, we describe a mechanism for tissue-wide cell–cell communication that requires neither cell–cell contact nor long-range diffusion of soluble factors. Mammary epithelial cells that have bypassed the *p16* arrest barrier (vHMEC) induce protumorigenic phenotypes in human mammary fibroblasts (HMFs) through activin A signalling [20]. In turn, secretions from HMFs promote the motility of the vHMECs. These observations suggest that even soluble factors with degradation times too short to survive diffusion throughout a tissue can still contribute to tissue-wide cell–cell communication if carried by motile cells before secretion.

### 4.2. Techniques in mathematical and computational modelling

To understand what models can be practically analysed through mathematical data analysis, it is helpful to be familiar with the kinds of derivations, equations and simulations that are currently applied to study population dynamics. Current techniques in theoretical analysis and computer simulation allow for more sophistication than displayed by the basic propositions and derivations presented in §3. We highlight a few examples. Readers who wish to review introductory material on EGT before investigating these refinements are referred to [21,22].

#### 4.2.1. Structured populations

In all of the models in §3, there is an assumption of thorough mixture. Even in those models where the cells are sessile, every cell is effectively in communication with every other cell because the signalling factors that disperse throughout the surrounding medium are themselves well-mixed. In structured populations, on the other hand, a particular cell does not interact in the same way with every other cell in the system. For example, consider a sessile cell that communicates with other cells by secreting chemical factors. Its communication with nearby cells might differ from its communication with distant cells if some of the signalling factors it secretes degrade quickly and can only reach nearby cells. Adding structure to a population can qualitatively change the population's dynamics. For example, *Escherichia coli* cells can secrete toxins that antagonize other cells, and the outcome of competition depends on whether strains are co-cultured in a well-mixed environment or on soft agar plates [23,24]. As shown using computer simulations [25], spatial structure can promote coexistence. Additionally, spatiality can make some transitions between steady-state population compositions more realizable. A well-mixed population might exhibit bistability; for example, being stable when purely composed of cell type *x*, and also being stable when purely composed of cell type *y*. A large population initially composed of cells of type *x* will restore itself even after stochastic contamination by a small number of cells of type *y*. However, in a population broken up into partially isolated niches, even the appearance of a handful of cells of type *y* may move the *local* population composition into the basin of attraction of the steady state consisting purely of cells of type *y*. After establishing a foothold, subpopulation *y* can then expand to take over the entire population [26]. Reviews of mathematical techniques for modelling spatial populations include the previous studies [26,27]. Finally, it is important to be aware that dynamics equations derived from *non*-spatial models can sometimes accommodate population dynamics from spatially structured systems [28,29]. Thus, discovering agreement between equations from a non-spatial model and data does not imply that the data come from a non-spatial system. Non-spatial analysis can serve as a first step in analysing coarse features of a biological system before spatial effects are explicitly taken into account.

#### 4.2.2. Agent-based (individual-based) models

Another limitation of the derivations in §3 is that they generate differential equations that track only *aggregate* population dynamics without providing time-course information about the events (e.g. cell division, death, signalling, etc.) in which any *particular* cell participates. Agent-based models (ABMs) address this limitation by simulating the behaviour of each individual cell (or cluster of cells). These models have been applied in the study of cancer [30], for example to describe the expansion of glioblastoma multiforme [31] and ductal carcinoma *in situ* [32]. In addition to domain-specific applications, ABMs can also be used to develop general principles of social evolution. For example, Aktipis [33] used an ABM to study contingent movement. In her model, agents that measured insufficient production of public goods from their neighbours would ‘walk away’ to find other neighbours. This helped to protect ‘cooperators’ from being exploited by freeloaders. Unlike in a corresponding well-mixed system, the cooperators could avoid being driven to extinction. Pacheco *et al.* [34] later used the phrase ‘active linking’ to describe the ability for agents to choose with which other agents to interact. They found analytic expressions for conditions for the promotion of cooperation among agents that perform active linking [34].

#### 4.2.3. Stochasticity

A third limitation in using the replicator equations in equations (1.1) and (1.2) is that they are deterministic. In finite populations, stochastic fluctuations are not necessarily ‘averaged out’. Abrupt, irregular jumps in population number can occur when a particular cell happens to die, or when two cells happen to collide at a particular time, rather than immediately before or after. While stochastic effects might be most familiar to biologists investigating fluctuating chemical reaction kinetics and gene-expression noise [35], stochasticity is also anticipated to contribute to the dynamics of multicellular tissue systems [36]. Stochastic extensions to traditionally deterministic EGT dynamics can be expressed and analysed using stochastic differential equations [37], numerically simulated using the Gillespie algorithm (kinetic Monte Carlo) [38,39] and interpreted in terms of a selection ‘temperature’ analogous to statistical mechanical temperature [40].

#### 4.2.4. Dynamic parameters

As another limitation, we point out that the models in §3 were used to obtain dynamics equations with constant parameter coefficients. In these models, the fitness of a cell type can vary with population composition, but the way in which the fitness varies with population composition is constant in time. For example, a population of two cell types might start out with a composition of *p_x_* = 0.3, *p_y_* = 0.7 and a fitness for cells of type *x* of *f_x_* = 2 d^−1^. This value might change to *f_x_* = 3 d^−1^ as the population composition changes to *p_x_* = 0.6, *p_y_* = 0.4. Nonetheless, the dependence of fitness on composition is constant in the sense that *f_x_* will always be 2 d^−1^ at each time the population has the composition *p_x_* = 0.3, *p_y_* = 0.7 and 3 d^−1^ at each time the population has the composition *p_x_* = 0.6, *p_y_* = 0.4. This constraint seems artificial given that a population of cells evolving in total number and composition might alter its environment, including the mechanical features that influence cell motility and the frequency of cell–cell encounters, as well as the chemical properties of the medium that carries molecular signals. In a more general approach, not only could cell numbers and cell fitnesses be dynamic, but intercellular signals and their meanings [41] and consequences for fitness [42] could also evolve over time. This means that the parameters *A*, *B*, *C* and *D* in equations (1.1) and (1.2) might vary.

### 4.3. Experimental systems

In addition to ensuring that a set of propositions can be theoretically analysed, it is necessary to ensure that a biological system can be experimentally manipulated and observed. Table 2 lists examples of techniques that can be used to observe the dynamics of interacting populations *in vitro* and *in vivo*. In addition to traditional tissue culture techniques [43], more recently developed 3D techniques can be used to investigate aspects of heterotypic tissue structure *in vitro* [45]. Recently, microfabrication techniques from engineering have been applied to develop silicon microecologies that allow cells to explore designed spatial habitat structures and chemical gradients [46,47], and nanofabrication techniques have been applied to develop nanopipettes that can probe and manipulate the molecular state of individual cells and individual organelles in a non-destructive fashion [48]. Owing to the relative difficulty and expense associated with *in vivo* experiments in preclinical and clinical settings, models are often validated in mechanistic detail *in vitro*, with a subset of predictions validated *in vivo* using time-course imaging, fluid biopsy and histology.

**Table 2.** Comparison of experimental population technologies for investigating cellular populations (SPIO, superparamagnetic iron oxide labelling; RH, ionizing radiation hazard limits frequency of imaging; DOSI, diffuse optical spectroscopic imaging; MRI, magnetic resonance imaging; PET, positron emission tomography; CT, computed tomography; CTC, circulating tumour cell; asterisk (*) denotes capability might be limited or not yet widely demonstrated to provide quantitative accuracy; dagger (†) denotes resolution at depth in tissue—see [49] for discussion of dependence of spatial resolution on depth). Order-of-magnitude resolution estimates are based on assuming densely packed cells with a cell diameter of ~10 microns.

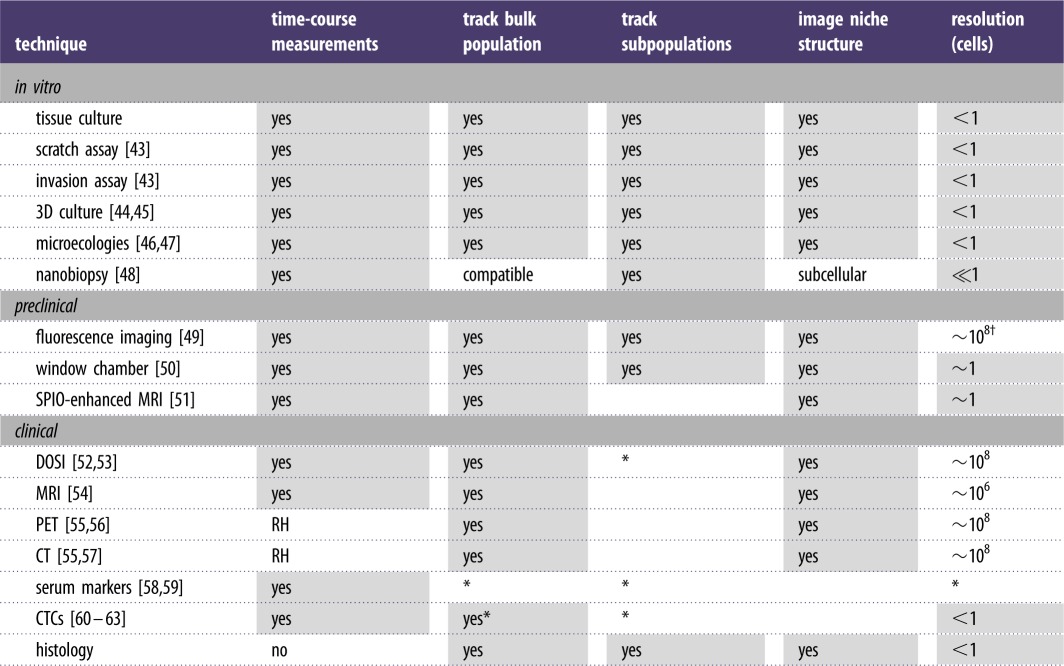

## 5. Discussion

In the previous manuscript [1], we presented a method for associating fitting equations with time-course measurements of dynamic populations. We followed up in this manuscript by demonstrating how interpretations could be associated with fitting equations. Taken together, these two manuscripts show how connections can be established between models and equations and between equations and data. These steps together, not just the establishment of connections between equations and data alone, constitute an idealized form of physical modelling.

A single set of differential equations can be derived from a variety of sets of starting assumptions. We outlined some considerations that can be used to choose interpretations, mathematical and computational techniques, and experimental systems to include in an investigation. In this section, we describe opportunities for advancing the application of game theory in biology. In §5.1, we propose that *in vivo* imaging methods need to be engineered to resolve cell-type differences. In §5.2, we argue for expanding our concept of biological systems beyond the concept of ‘systems of cells’. We conclude in §5.3 by describing the possibility that a deeper understanding of dissipative structures might eventually allow us to identify and to understand the essential features of biological population systems.

### 5.1. Engineering clinical and *in vivo* monitoring

We mentioned that one reason for combining *in vitro* and *in vivo* studies is the relative difficulty and expense of performing preclinical and clinical trials. Table 2 illustrates another reason that necessitates *in vitro* experiments. A variety of *in vitro* techniques can be used to perform repeated time-course measurements of the dynamics of individual subpopulations and to image multicellular spatial arrangements with single-cell, or even subcellular, resolution. However, *in vivo* methods tend to lack at least one of these features. Window chamber experiments provide an exception. Unfortunately, this technique cannot be applied in clinical trials for ethical reasons. Ethical concerns also limit the clinical use of fluorescent reporters that allow different cell types to be distinguished using whole-animal fluorescence imaging and window chamber methods.

A capability important for validating game theoretical models is the ability to distinguish between cellular subpopulations in repeated, non-destructive time-course measurements *in vivo*. Possibilities for addressing this need without the use of genetic constructs include multiplexing contrast agents based on uptake, exclusion, surface binding and periplasmic binding. For example, if two contrast agents could be attached to two different antibodies, MRI could then be used to measure the spatial distributions of different targeted cell surface receptors. To further distinguish between cell types, one might multiplex different imaging methods. For example, the spatial distribution of surface markers could be supplemented by knowledge of the spatial distribution of haemoglobin and water content in a tissue, which can be obtained using diffuse optical spectroscopic imaging [52,53].

### 5.2. Beyond a focus on the cell

In §4, we gave examples of refinements to traditional EGT models that might be necessary for modelling the dynamics of cell numbers with accuracy and detail. In this subsection, we argue that, even if every change in cell number could be accurately described, we might still lack a comprehensive understanding of cancer systems because there is more to cancer biology than *cell number* and *the cell*.

In EGT, a model is typically applied to determine how the net replication rate of a particular cell type changes in response to changes in the demographic composition of the overall population. However, net replication rates are only one phenotype of biological interest. For example, it has been recognized that the consequences of epithelial–stromal interactions include alteration not just of proliferation and death rates, but also of cellular arrangement, genomic instability, vascularization and invasiveness [64,65]. Focusing on cell number alone can, in principle, lead to clinical strategies that conflict with strategies obtained from a broader perspective. Suppose that a subpopulation of cells were included as an explicit cell type in an EGT model. This could be because they were relatively ‘aggressive’. It might not suffice merely to decrease the number and population fraction of these cells. If these goals were achieved, but, at the same time, these cells increased their aggregate output of, say, cachexia-associated cytokines, then overall outcome might nonetheless be disease progression. For a more complete understanding of a tissue system's biology, it is important to supplement knowledge of the time variation of population fractions with a model that describes the consequences of these changes for the phenotypes that cells display. When we apply such models, we might discover that we can target phenotypes associated with disease without needing to completely eradicate ‘abnormal’ cell populations.

In applications of EGT to cancer, models are often applied by identifying the ‘agents’ or ‘replicators’ with cells. The analysis techniques in this tutorial are not specific to the time and length scales of cells. The mathematical methods in this tutorial are actually often first introduced in undergraduate chemistry courses as tools for analysing the kinetics of sub-microscopic chemical reactions [66]. Even though the cell is sometimes regarded as the fundamental unit of life (on the Earth), it would probably be valuable to study interactions between tissues, organs, organisms and organism populations. One emerging perspective regards cancer metastasis as a diaspora of cells migrating in search of high-quality habitats [67]. Pienta *et al.* [67] have proposed the use of ‘attracticides’, which are low-quality habitats disguised using cues typically associated with high-quality habitats. These cues could be mechanical and chemical. Thus, the interactions that would be useful to incorporate explicitly in a mathematical model would not be exclusively limited to interactions between cells, but would also include interactions between cells and the spatially heterogeneous chemical and mechanical properties of their environments. Macklin *et al.* [32] recently incorporated the mechanical properties of dying cells into a patient-calibrated model to understand the relationship between tumour size and time and the relationship between calcified tumour size and time in ductal carcinoma *in situ*. They found that necrotic cells provided a mechanical stress relief throughout the bulk of the tumour so that only the proliferation of cells near the leading edge would contribute to increasing the tumour's volume. The linear relationships between tumour size (and calcified tumour size) and time, as well as the ‘error’ between the two tumour sizes in their ABM, might someday assist the planning of surgical margins for lumpectomies. The agents in a game theoretic analysis need not be confined to a single tissue or organism. Orlando *et al.* [68] modelled the combined evolution of tumour cell number and treatment choices that an oncologist could make. In this example, one of the interacting agents (the oncologist) is an organism entirely separate from the patient.

### 5.3. Developing a fundamental physics of living systems

While adding detail to the assumptions of a model can yield more realistic simulation results, combining the refinements in §4 potentially renders models more complicated to interpret and use. It would be helpful to command a fundamental understanding of biology that allowed us to pinpoint minimal propositions sufficient for describing the essential features of interacting population systems. The physics of dissipative structures might eventually provide such understanding. In this final subsection, we summarize this perspective, which relates to ideas discussed by Goldenfeld & Woese [42], and then comment on the possibility of proceeding with physical biological research while a physics of living systems continues to develop.

Physicists sometimes regard living systems as examples of ‘dissipative structures’. To define this phrase, we first describe the concept of a ‘heat death’. Consider an isolated collection of dynamic objects (e.g. protons, neutrons, electrons, photons, etc. in a perfectly sealed and isolated box), which we call a universe. Over time, the contents of the universe manifest an equilibrium mixture. Fluctuations can still locally increase or decrease the concentration of matter and energy, but these fluctuations then dissipate into the background of homogeneity. This inevitable condition is called a heat death.

The contents of the universe might initially be arranged in a configuration that qualitatively differs from a heat death, for example with all particles initially concentrated near one corner of the box. As the contents of the universe redistribute themselves over time, a subset of the particles might temporarily be arranged in a pattern that exchanges energy and matter with its surroundings. Specifically, this pattern might maintain a structure that is *temporarily* stable or cyclic and *locally* distinct not only from the state of heat death, but also from a state of approaching heat death. This is a dissipative structure. A dissipative structure can itself be composed of smaller, interacting dissipative structures.

In principle, dissipative structures include living organisms. Thus, it has been hypothesized that improved understanding of dissipative systems will suggest relationships involving the dynamics of the substructures of biological systems. Based on this perspective, we might study dissipative structures in hopes of, as an extreme example, developing intuition about the minimum number of cell types that must interact to perform a particular function. Such knowledge might assist in the narrowing down of choices of models and experimental co-culture systems to investigate.

Table 3 compares models and principles that have contributed to breakthroughs in Newtonian mechanics, quantum mechanics and equilibrium statistical physics. The column describing non-equilibrium statistical physics depicts a field that is not yet fully developed. An empirical approach remains worthwhile to pursue. In introductory physics, the quantum mechanical descriptions of rigid objects, strings and surfaces, as well as the forces between them, are not usually presented. Nevertheless, Newtonian mechanics is widely applied to describe the motion of, for example, masses straddled across pulleys, because the origins of tension, static friction, kinetic friction, normal force, gravity and fluid drag need not be understood to complete **F_NET_** = *m***a** calculations. A physics of living systems might one day mature from the physics of dissipative structures, but even now we are optimistic that interdisciplinary collaboration, with physical scientists and biologists modelling mechanisms of cell–cell interaction, with biologists and clinicians identifying tissue systems with relevance to disease, with physicists and engineers improving methods to image cellular subpopulations *in vivo*, and with biologists, clinicians and patient advocates developing treatment plans, will realize game theory's potential to improve our understanding of cancer and its control.

**Table 3.** Comparison between modern development of the physics of living systems and other far-from-equilibrium systems with previous developments in Newtonian mechanics, quantum mechanics and equilibrium statistical physics.

Newtonian mechanics	quantum mechanics	equilibrium statistical physics	far-from-equilibrium statistical physics
Kepler's laws	Bohr atom	Carnot cycle classical thermodynamics	Belousov–Zhabotinsky reaction Jarzynski equality
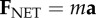 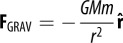	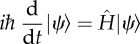	microstates equal probability postulate 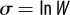 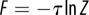	principal properties of emergent relationships in dissipative systems? *ab initio* relationships involving modularity, scales, topologies?
Neptune	transistor	Bose–Einstein condensate	?

## Supplementary Material

Supplemental text and table
