# Supplementary material for: Evolutionary game theory for physical and biological scientists. II. Population dynamics equations can be associated with interpretations
Source: Interface Focus. 2014 Aug 6;4(4):20140038. doi: 10.1098/rsfs.2014.0038 (PMC4071514; doi:10.1098/rsfs.2014.0038)
Supplement: Supplemental text and table [file rsfs20140038supp1.pdf]

Supplemental materials

Evolutionary game theory for physical and biological scientists: II. Population dynamics equations can be associated with interpretations

David Liao and Thea D. Tlsty

Department of Pathology, University of California San Francisco, San Francisco, CA 94143

Sections, tables, and equations are numbered as follow.

	Sections	Tables	Figures	Equations
Main manuscript	1-5	1-3	1-4	1.1-3.13
This supplement	6	4	—	6.1-6.5

Contents

6. Steady-states of evolutionary dynamics and solutions of comparative statics 2
6.1. Steady-state in evolutionary game theory 2
6.2. Solutions in comparative statics 3
Supplemental table 5

6. Steady-states of evolutionary dynamics and solutions of comparative statics

It is sometimes claimed that competing populations of interacting, true-breeding replicators described using evolutionary dynamics will achieve the same solution as a related set of rational agents choosing strategies to optimize their utility as described using comparative statics. This section provides an intuitive understanding for this claim. The purpose of this section is not to provide a rigorous analysis; indeed, stability is not seriously addressed. The primary goal is to demonstrate that the sense in which evolutionary game theory and comparative statics are said to achieve the same solution has more of a flavor of visual similarity in notation between two analyses than any sense that would suggest that cellular replicators possess the quick-thinking, rational, intelligence of human agents. It is important to remember that while cellular populations and rational agents arrive at related solutions, the cellular populations approach their solution over an infinite number of generations while the rational agents in comparative statics may be able to identify and adopt their optimal strategies in an instant before they interact.

6.1. Steady-state in evolutionary game theory

We first demonstrate a condition describing steady-state in evolutionary game theory and then compare it to a condition describing a "solution" in comparative statics. The population fractions p_x, p_y, . . . , p_z evolve while $\mathrm{d}p_x/\mathrm{d}t$, $\mathrm{d}p_y/\mathrm{d}t$, . . . , $\mathrm{d}p_z/\mathrm{d}t$ are non-zero. If the population approaches a stable composition, the time derivatives become small, and eventually, arbitrarily close to zero. To describe this kind of equilibrium, we set the time derivatives in equation 5.6 from the supplement accompanying the preceding manuscript [1] and analogous expressions equal to zero. For example, setting the rate of change of the fraction p_x

$$\frac{\mathrm{d}\,p_x}{\mathrm{d}t} = p_x\left[f_x - \left(f_x p_x + f_y p_y + \cdots + f_z p_z\right)\right] \tag{6.1}$$

to zero and assuming that the subpopulation of type x has not vanished ($p_x \neq 0$) yields the condition

$$f_x = f_x p_x + f_y p_y + \cdots + f_z p_z \tag{6.2}$$

Because an analogous condition is obtained for each of the remaining variables, p_y . . . p_z, all the fitnesses are equal to the same quantity, $f_x\, p_x + f_y\, p_y + . . . + f_z\, p_z$, and thus, equal to each other

$$f_x = f_y = \ldots = f_z \tag{6.3}$$

In a steady state, the fitnesses are equal for all populations that have not yet vanished. Because these populations expand or shrink at the same rate, their relative proportions remain constant. To be clear, equation 6.3 only describes a steady-state. We have not established that the steady-state is stable. This would require demonstrating that this steady-state population composition would tend to restore itself small disturbance. However, for the purposes of developing an intuitive understanding of how the solutions for evolutionary dynamics and comparative statics can be "the same," the partial description of the steady-state in equation 6.3 suffices.

6.2. Solutions in comparative statics

To identify a mathematical expression characterizing "solutions" to comparative statics, consider a two-way game described in Table 4. The agent whose outcome we track ("focal individual") rolls dice to choose randomly among strategies s_X, s_Y, . . . s_Z (column at left) with likelihoods p_x, p_y, . . . p_z, respectively. The focal individual confronts a second agent who also chooses randomly from among the same strategies, s_X, s_Y, . . . s_Z (row at top) with likelihoods $\tilde{p}_x, \tilde{p}_y, \ldots \tilde{p}_z$. The matrix elements enumerate the payoffs that the recipient achieves in different scenarios. For example, the recipient earns payoff A_{xy} if she chooses strategy s_X while the other player chooses strategy s_Y. This event occurs with frequency $p_x \tilde{p}_y$ and so contributes a quantity $p_x \tilde{p}_y A_{xy}$ to the payoff

$$\begin{aligned}\langle U \rangle = \quad & p_x A_{xx} \tilde{p}_x + p_x A_{xy} \tilde{p}_y + \cdots + p_x A_{xz} \tilde{p}_z \\ & + p_y A_{yx} \tilde{p}_x + p_y A_{yy} \tilde{p}_y + \cdots + p_y A_{yz} \tilde{p}_z \\ & + \cdots \\ & + p_z A_{zx} \tilde{p}_x + p_z A_{zy} \tilde{p}_y + \cdots + p_z A_{zz} \tilde{p}_z \end{aligned} \tag{6.4}$$

averaged over all scenarios, weighted according to scenario likelihoods. A solution or "equilibrium" in comparative statics occurs when each player adopts the same strategies and the same likelihoods of choosing among those strategies, and, furthermore, no unilateral adjustment to the likelihoods is able to increase the average payoff $\langle U \rangle$ of either player. The first condition means that $p_x = \tilde{p}_x$ (and likewise for the other strategy likelihoods). Equation 6.4 can now be more compactly expressed as

$$\langle U \rangle = p_x f_x + p_y f_y + \cdots + p_z f_z \tag{6.5}$$

using $f_x = A_{xx} p_x + A_{xy} p_y + A_{xz} p_z$ and analogous relationships for $f_y \ldots f_z$. The second condition states that no player can achieve a higher average payoff by unilaterally adjusting her probabilities. This implies that the quantities $f_x, f_y, \ldots f_z$ are equal to each other. If they were unequal, it would be possible to increase the average payoff $\langle U \rangle$ by shuffling probability from a strategy with a lower value of f to another strategy with a higher value of f.[1] Thus, both the analysis of steady states in EGT population dynamics models and the analysis of equilibria in comparative statics lead to the same symbolic expression, $f_x = f_y = \ldots = f_z$. The solution condition for evolutionary dynamics and the solution condition for comparative statics are "the same" in a sense that in no way requires cellular replicators to exercise humanly sophisticated intelligence. Thus, the claim that evolutionary dynamics and comparative statics have the same solution should not be interpreted as rendering EGT inapplicable for modeling biological systems.

Supplemental reference

[1] Liao D, Tlsty TD 2014 Evolutionary game theory for physical and biological scientists: I. Training and validating population dynamics equations *Interface Focus* **4** 20140037 (doi:10.1098/rsfs.2014.0037)

---

[1] We are ignoring the possibility of an "edge" situation in which one or more probabilities have already been exhausted and can no further be decreased or where one of the probabilities has already equaled unity and can no further be increased.

Supplemental table

Table 4. Payoff for a focal individual in different scenarios. In each scenario, one strategy is adopted by the focal individual, one strategy is adopted by an agent from the environment, and the two agents interact.

		**Strategy of agent from the environment**			
		s_x	s_y	…	s_z
**Strategy of focal individual**	s_x	A_{xx}	A_{xy}	…	A_{xz}
	s_y	A_{yx}	A_{yy}	…	A_{yz}
	⋮	⋮	⋮	⋱	⋮
	s_z	A_{zx}	A_{zy}	…	A_{zz}
